# Various vaccine platforms in the field of COVID-19

**DOI:** 10.1186/s43088-022-00215-1

**Published:** 2022-03-07

**Authors:** K. Savina, Rakhy Sreekumar, V. K. Soonu, E. J. Variyar

**Affiliations:** Department of Biotechnology and Microbiology, Dr. Janaki Ammal Campus, Kannur University, Kannur, Kerala 670661 India

**Keywords:** COVID-19, Vaccines, Infection, LAV, RBD

## Abstract

**Background:**

With the emergence of Corona virus Disease-2019, a novel worldwide health disaster is threatening the population. The WHO declared COVID-19 as a pandemic in December 2019, when it first surfaced in Hunan seafood market in Wuhan, South China, and quickly spread far and wide. Different corona virus variants are currently causing concern all across the world.

**Main body:**

It has become critical for our scientists to develop a viable method to prevent infection or the pandemic from spreading globally. Antiviral medicines, oxygen therapy, and immune system stimulation are all used to treat the condition. SARS-CoV-2 undergoes mutation and due to evolutionary pressures, different mutant strains caused various symptoms in different geographical regions and the epidemic is spreading and becoming more fragile, posing a greater risk of mortality. Vaccines are tools to increase our immunity as a precaution, and increasing the global immunization rate can help improve the situation. Recent developments in the field of vaccine platforms are discussed here.

**Short conclusion:**

Vaccines are of highest priority to control and eradicate the viral infectious disease COVID-19 more than any other protective solutions. A number of mutations have occurred and some variants such as alpha, beta, gamma, and delta, and it has now progressed to the new version Omicron, which is a variant of concern. Booster doses are anticipated to function as a barrier to the capacity of the most recent known variety, and more research is needed to determine how effective they will be. This page discusses various technologies employed in the field of COVID-19 vaccine, as well as potential barriers and recent developments in this field.

## Background

SARS-CoV-2, the virus that causes COVID-19, has spread around the world since December 2019. As it undergoes mutation and due to evolutionary pressures, the epidemic is spreading and becoming more dangerous, posing a greater risk. Several medicines are now being explored in clinical trials as potential repurposing candidates, however only remdesivir has been approved for the treatment of COVID-19 to date [1]. Even up with the latest approval of the antiviral drug remdesivir, as well as the Emergency Use Licensing of monoclonal antibodies against S protein, bamlanivimab and casirimab/imdevimab, efficient and safe COVID-19 vaccines are indeed urgently needed, not only to stop the virus's spread but also to revive social and economic activities through large scale immunization. Pfizer and BioNTech's mRNA vaccine received Emergency Use Authorization recently, which may provide a road ahead, but long-term immunity must still be monitored, and a number of alternatives are still being developed [2]. Even though a completely effective treatment is not yet found, researches are still ongoing to find a stable therapeutic solution to impair the infection or to decrease the spreading [3]. Vaccines are of highest priority to control and eradicate the viral infectious disease COVID-19 more than any other protective solutions. Various technology platforms are under investigation and advancement to create a competent vaccine against COVID-19 [4].

Multiple viral variants have evolved and grown prevalent in numerous nations since January 2021. The introduction of these virus variants Alpha, Beta, Gamma, and Delta was responsible for new waves of infections all across the world [5]. The advent of highly mutated (SARS-CoV-2) variant Omicron (B.1.1.529), which is a variant of concern (VOC) is now sparking fear around the world [6]. As of January 2022, WHO reports 334 vaccines are in trial of which 140 are in clinical phase and 194 in preclinical phase [7]. Vaccines in trials include protein-based or subunit and virus-like particle vaccines, virus-based or live attenuated and inactivated vaccines, and innovative gene delivery techniques such as viral vector or replicative and non-replicative and nucleic acid or DNA and RNA vaccines [8] have all been used in the development of vaccination candidates. Several big clinical trials evaluating the COVID-19 vaccines, clinical effectiveness and safety have generated promising results, despite the fact that many of these vaccines are still in pre-clinical testing. Booster doses, according to recent findings, raise neutralizing-antibody levels, which will likely act as a barrier to latest known variant’s capacity to avoid these antibodies. However, how effective these doses will be against this virus strain is unknown. People who have been exposed to the protein of the SARS-spike CoV-2 virus on multiple occasions, whether through infection or a booster dose, are more likely to have neutralising antibodies against the latest variant [9, 10].

The inability to validate and target the correct vaccination platform technologies, as well as the failure to create long-term immunity and the inability to quiet cytokine storm, are all significant roadblocks in the development of the COVID-19 vaccine. In addition to traditional vaccination forms, developing nanotechnology-based vaccine techniques are very versatile and contribute to vaccine development speed [11]. Since COVID-19 vaccine is a hot and relevant topic in this pandemic situation, we took an initiation to narrate the article. The assessment and use of currently available vaccines are in critical for a timely response to the current pandemic. We have reviewed various platforms in the field of COVID-19 vaccines although clinical trials to assess the efficiency of these vaccines are ongoing. This review describes the structure of SARS-CoV-2 and different vaccine platforms used in the development of COVID-19 in detail.

## Main text

### Structure of SARS-CoV-2

SARS-CoV-2, a beta corona virus belongs to the ‘Coronavirdae’ family with a single-stranded, positive-sense, and enclosed RNA genome ranging in size from 26.5 to 31.7 kb [12]. The spike (S), nucleocapsid (N), membrane (M), and envelope (E) proteins are the four important structural proteins encoded. The S protein promotes viral uptake into host cells by binding to the host receptor, angiotensin-converting enzyme 2 (ACE2) [13]. The S1 subunit of S protein contains the receptor binding domain or RBD which facilitates the cell attachment, the S2 component, on the other hand, is accountable for mediating the fusion of the virus's membranes with those of the host cell [14]. Nucleocapsid (N) protein is the second structural protein that responds to the host cell and participates in the replication cycle [15]. The M protein is a three- transmembrane domain transmembrane glycoprotein. It is important in viral assembly and attaches to the nucleocapsid [16]. The final structural protein, the envelope (E) protein, which is the smallest of all structural proteins, with 76–109 amino acids and made up of hydrophobic viroporins [17]. Viroporins have a hydrophobic phospholipid coating on the outside [18]. As a result, the corona virus family's outer membrane possesses a lipid layer, which aids in communicating with the host cell [19].

The genomic organisation of SARS-CoV-2 consists of 13–15 open reading frames (ORFs), 12 of them are functional and enclose 11 protein-coding genes and 12 expressed proteins [20]. Once it enters the host cell, there occurs viral protein translation immediately with the help of a 3′-poly (A) tail and a 5′-cap. The transcriptional regulatory sequences are arranged sequentially with highly structured untranslated regions (UTRs) along a standard 5′–3′ path [21]. ORFs 1a and 1b, encode the replicase polyproteins 1a and 1ab respectively (PP1a and PP1ab). The non-structural proteins (NSPs) 1–16, that makes up the replicase complex, are encased in polyprotein 1ab, which is among the biggest polyprotein. Key structural proteins S, E, M, and N are encoded at the virus genomic RNA's 3′ end. These proteins are crucial for viral entrance, membrane fusion with the host body, and the integrity of mature viral structures [22–24]. Accessory proteins are considered to have further functions which are implicated in pathogenicity through modulation of the interferon signalling system even if not necessary for replication [25].

**Fig. 1:**
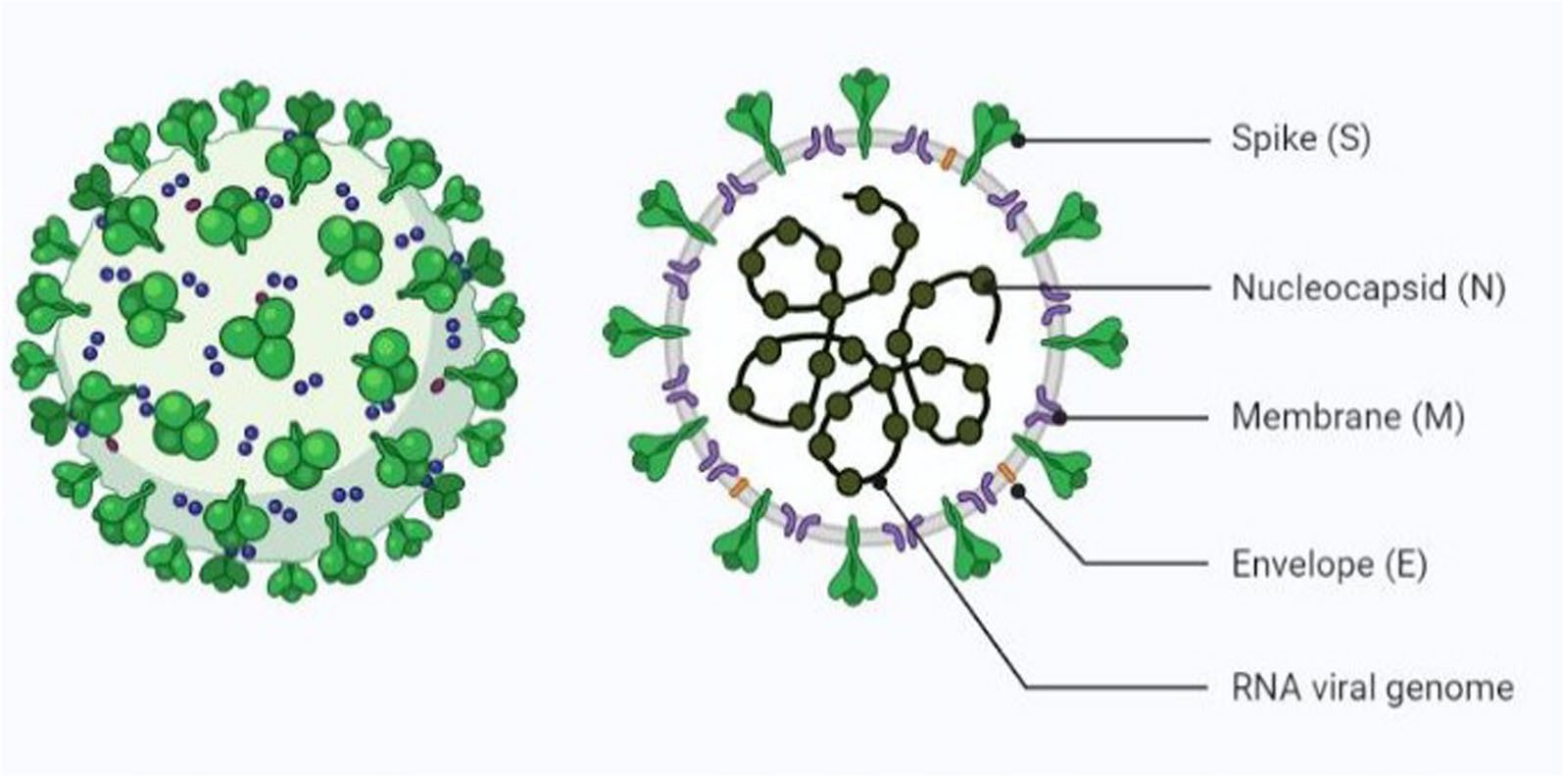
SARS-CoV-2 structure (Reprinted from “Human Coronavirus Structure”, by BioRender, August 2020, retrieved from https://app.biorender.com/biorender-templates/figures/5e99f5395fd61e0028682c01/t-5f21e90283765600b08fbe9d-human-coronavirus-structure Copyright 2022 by BioRender.)

A set of nested subgenomic RNAs (sgRNAs) is used to translate structural and auxiliary proteins, beginning with negative-sense RNA intermediates. NSPs are also important in the pathogenesis of virus, as they play a role in transcription regulation at early stage, gene transactivation, antiviral evasion, immunomodulation, and virus replication and assembly [26–29] (Fig. 1).

### Different platforms used for the development of COVID- 19 vaccines

**Fig. 2:**
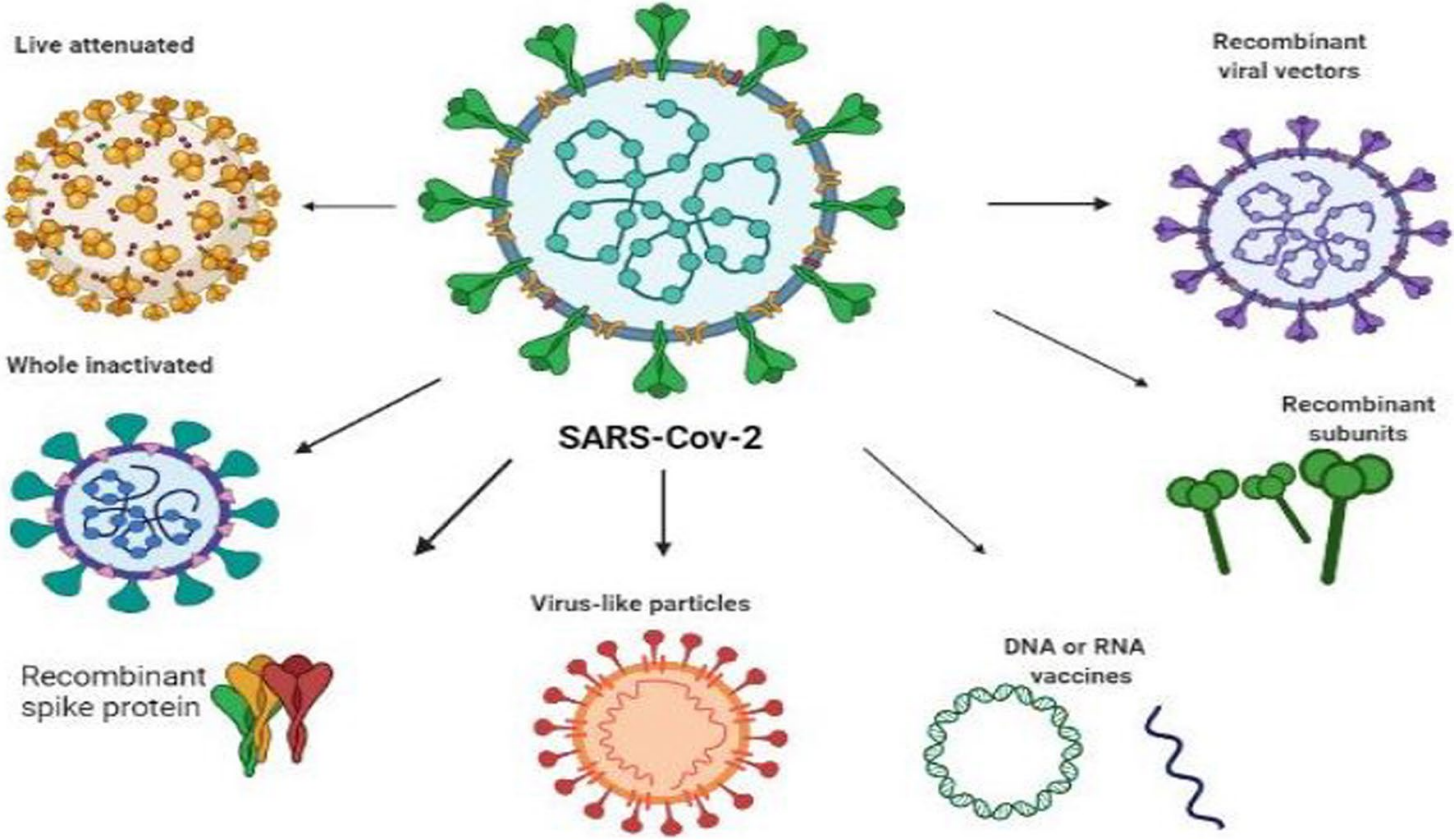
Various approaches of SARS- CoV-2 viral vaccine development (Reprinted from “Approaches to Viral Vaccine Development”, by BioRender, February 2020, retrieved from https://app.biorender.com/biorender-templates/figures/5e99f5395fd61e0028682c01/t-5e6256cdd7f17b0088a993d3-approaches-to-viral-vaccine-development Copyright 2022 by BioRender.)

COVID-19 vaccine was developed on a variety of platforms. Available vaccine platforms are virus-based (live attenuated and inactivated), protein-based (subunit and virus-like particle) and novel gene delivery strategies such as nucleic acids (DNA and RNA) and viral vectors based (replicative and non-replicative) (Fig. 2).

#### Virus based vaccines

##### Live attenuated

The live attenuated vaccine (LAV) requires genetic manipulation to develop low-replication variants of the virus that cannot cause disease but can elicit a similar immune response to that seen in natural infections. It has been linked to genetic instability and the presence of residual virulence [30]. LAV has a simple production procedure that uses well-established Vero cells, which have been used for the research of vaccines for almost 40 years and were now the most widely accepted cell lineage for vaccine development by regulatory agencies. LAVs have also been shown to flourish and infect microcarrier beads without affecting production output at large-scale fermenters up to 6000 L, in serum-free conditions [31].

##### Inactivated

To inactivate the virus, these vaccines uses some chemicals such as psorlens, hydrogen peroxide ascorbic acid, ethylenimine derivatives, β-Propiolactone, formaldehyde or physical methods like UV treatment, heat gamma irradiation etc. or the combination of the both. For inactivation of licensed human viral vaccines, formaldehyde and, β-Propiolactone are widely used in the last decades [32]. The virus can be grown in cell culture using Vero cells and inactivated using chemical methods to make SARS-CoV-2 inactivated vaccines [33, 34]. Inactivated vaccines are part of the standard viral vaccination technique. Because these vaccines contain many antigenic components, they have the ability to elicit a wide range of immune responses [35]. In compared to live-attenuated vaccinations, they are said to have less reactogenicity and are associated with lesser immune responses. For inactivated vaccines to be effective, multiple inoculations and powerful adjuvants may be required. Even while the well-established and streamlined development approach is beneficial, it does entail the handling of a live virus [36].

#### Protein-based vaccines

##### Subunit vaccines

Purified immunogenic proteins or peptides make up subunit vaccines [37]. The majority of CoV subunit vaccines target the Spike protein, particularly its RBD which is highly immunogenic [38]. Vaccination targets include viral structural proteins such as small envelope protein E, envelope spike protein S, nucleocapsid protein N, and matrix protein M [39]. By cleaving the spike protein and permitting the S2 subunit to fuse to the cellular membrane, the serine protease, TMPRSS2 plays a role in the process [40]. SARS-CoV RBD antibodies cross-react with the respective protein, and the resultant antisera neutralises the virus, shows that a vaccination targeting the S protein domain could be successful in preventing COVID-19 [14]. The profusion-state of RBD in subunit S1 is responsible for binding to ACE2, whereas the cleavage site in subunit S2 is required for viral and cellular membrane fusion [41]. The S protein of full-length i.e., S1, S2 subunit and RBD, proteins were identified as critical epitopes for generating neutralising antibodies as per computational analyses and studies on the viruses, SARS-CoV and MERS-CoV [42, 43].Antibodies against the RBD domain have early been shown protection against SARS and MERS-CoV infections, and the S1 epitope, containing both the RBD as well as N-terminal binding domain, NTD, has also been used to develop vaccines [44, 45]. A cluster of T cell epitope was discovered in the transmembrane part of the M protein, allowing the development of a significant cellular type of immune response against the SARS-CoV [46]. Novavax used Matrix-M adjuvant recombinant protein nanoparticle technology and the Sf9 system to develop the subunit vaccine candidate for SARS-CoV-2, the NVX-CoV2373 which comprises a trimeric full-length S-protein with the GenBank accession number MN908947 with nucleotides 21563–25384 that are missing the polybasic cleavage site, 682- QQAQ-685 was added to the S1/S2 junction to improve protease resistance, and K986P and V987P were added to enhance the stability of the recombinantly generated vaccine antigen [47] and might elicit immunological responses that were higher than those elicited by Matrix-M1 adjuvant and COVID-19 convalescent serum responses, both of which were biased toward a Th1 phenotype. The antigen in Clover Biopharmaceuticals' S-Trimer vaccine is a recombinantly generated homotrimer of the full-length S-protein [48]. A protein subunit vaccine, also known as an adjuvanted recombinant vaccine, is assembled of virus components that enhance the human immune system without incorporating virus particles into the body [49]. Russia has recently established recombinant adenovirus vectors of type 26 (rAd26) and type 5 (rAd5), both of which carry the SARS-CoV-2 Spike protein gene. It was established that lyophilised and frozen vaccines were reported as good and safe which could induce a strong cellular and humoral immune response [50].

##### Virus-like particles

Protein based vaccines consist of virus-like particles (VLPs), the recombinant proteins or supramolecular structures which may contain one or more copies of 10–200 nm nanoparticles assembling viral proteins [51]. Virus-like particles are created using structural proteins that have been recombinantly generated (VLPs). VLPs, the S protein of SARS-CoV-2, facilitate host cell fusion via ACE2 receptor binding and priming via TMPRSS2, unlike in subunit vaccines where VLPs seem to be unable to directly attach to B cell receptors to form the antibodies [52]. The VLPs of SARS-CoV-2, which are derived from genetically modified plants, have been shown to be effective in the production of neutralising antibodies [53].

#### Gene-based vaccines (GBVs)

GBV encloses RNA, DNA, and the viral vector platforms and also each of them contributes peculiar advantages and disadvantages.

##### DNA vaccines

DNA vaccines, which are generally made up of a plasmid vector that encodes a target vaccine molecule and can elicit longer period of cellular and humoral immunity, can be mass-produced in large quantities [54]. This type of vaccine does not require the use of live viruses and may be freeze-dried and stored for a long time even though in underdeveloped countries, a major issue like power outages can be faced where some vaccine batches become inoperable [36, 55]. Through DNA vaccines, immune responses are prompted against recombinant antigens encoded by genetically engineered DNA plasmids. On post vaccination, the cellular machinery of the host enhances plasmid-encoded genes expressions which results in foreign antigens synthesis and that can be presented and processed by molecules of both class I and IIMHC. The immune system is able to recognise these foreign antigens generated by the host, resulting in a complete and adequate immunization [56].

##### RNA vaccines

RNA- based vaccines invigorates the immunogen production via induction of both cellular and humoral immune responses. These vaccines also act on DNA based vaccine’s principle with an exception that there is no need of translocation to the nucleus for RNA transcription. They can be made from either self-replicating RNA or mRNA, mRNAs are generated with nucleoside modification to avoid the degradation, also with lipid nanoparticles like carrier molecule to allow RNA entry in to the cells [57]. BNT162b2, an mRNA vaccine candidate authorised in the United Kingdom, was the first RNA-based vaccination to be approved [58]. mRNA vaccines must be supplied to the host cell's cytoplasm for translation into antigenic proteins and is a promising replacement for traditional protein or whole virus vaccines because of its increased efficacy for producing an immune response, safety, alsoits quick and low-cost manufacture [59]. BNT162b1, a lipid-soluble nanoparticle formulation containing mRNA encoding the S protein RBD trimer, was produced by Pfizer and BioNTech [60].

##### Viral vector vaccines

Viral vector vaccines consist of an often attenuated recombinant virus, designed to encode sequence of an antigen for host cell delivery for high level endogenous production of that antigen and thus induces elevated levels of cellular and humoral immune responses. These vaccines grants increased capabilities of gene transduction because of the natural host cell infection ability of the viruses [61, 62] and these are of replicating or non-replicating [4]. They are constructed to transmit one or many antigens of your choice, as well as the capacity to load a big genome suggest that a wide range of vaccines might be developed [63].

###### Non-replicating

CanSino Biological Inc. and Beijing Institute of Biotechnology's Ad5 adenovirus vector vaccine, Ad5-nCoV, first vaccine candidate in the world to announce datas of clinical trials [64]. ChAdOx1-nCoV-19 (officially called AZD-1222) is a chimpanzee adenovirus (ChAd) recombinant vaccine produced by Oxford University and AstraZeneca that incorporates a codon optimized S protein gene added into the ChAd replication-defective mutant ChAdOx1. AZD1222 (AstraZeneca, UK) vaccination lowered SARS-CoV-2 viral load in the lungs and was protective against pneumonia, but it did not reduce viral levels in the upper respiratory tract in a challenge model of rhesus macaque. It is based on the ChAdOx1 vector, which encodes S protein and has a human tissue plasminogen activator (tPA) leader sequence flanked by poly A bovine growth hormone (BGH) signal sequence and cytomegalovirus promoter (CMV) [65]. These vaccines appeared to be safe, well tolerated, and resulted in increased levels of neutralising antibodies as well as significant ChAdOx1 nCoV-19-related side effects [66]. According to published findings from animal research, a single dose of the vaccine provided highly efficient cellular and humoral immunity. The viral load was seen much lower in vaccinated monkeys than in control monkeys after SARS-CoV-2 challenge, and pathology data revealed that in vaccinated rhesus monkeys, there was no antibody-dependent enhancement (ADE) impact and no pneumonia. A single inoculation of AZD-1222 in aged mice generated both humoral and cellular immunity however the extent was less than in young mice [67].

Gam-COVID-VacLyo (NCT04437875, renamed Sputnik V) is made up of two spike gene inserted adenoviruses, Ad5 and Ad26. The Gamaleya Research Institute in Russia developed it [68]. Sputnik V or Gam-COVID-Vac, a separate Russian vaccine candidate, contains frozen or lyophilized viral vectors of recombinant human rAd26 and rAd5 expressing the S protein. Clinical trials demonstrated that this heterologous vaccination was highly immunogenic and safe, eliciting robust cellular and humoral responses in all participants. RBD-specific IgGs were found in the majority of those who got this vaccination. Only 61% of subjects produced NAbs after receiving a single recombinant viral vector, but boosting with the additional recombinant vector boosted RBD-specific IgG titers. All of the participants produced SARS-CoV-2 NAbs after receiving the prime-boost, with titers that are comparable to COVID-19 recovered individuals. Common and expected symptoms like discomfort at the injection site, heat, asthenia, and headache were recorded after vaccination [50]. Even 7 days after the second injection, the Sputnik V vaccine exhibited a 90 percent efficacy rate. As a result, over 50 countries requested 1.2 billion doses of the vaccine, according to the Gamaleya Institute's second interim report [69]. Replication-defective viral vectors are widely used in non-replicating viral vaccination platforms. These are based on a weak strain of the common cold virus, which can infect human cells but not cause illness. Adenoviruses (Ad) are one of the most common non-replicating vectors designed to simulate natural viral infection and inducing the synthesis of target viral proteins within host cells [70]. Adenovirus type 5 (Ad5) is used to make non-replicating viral vectors, and the vast majority of these vaccines use RBD subunit or S protein of SARS-CoV-2 [71].

###### Replicating

The replicating vaccines, intranasal flu vectored, DelNS1-2019-nCoV-RBD-OPT1 (Beijing Wantai Biological Pharmacy) is now in phase 3 and VSV vectored rVSV-SARS-CoV-2-S/IIBR-100 vaccine (Israel Institute for Biological Research) is in Phase 2/3 of clinical trials, and measles-vector based TMV-083 (Institut Pasteur/Themis Bioscience/Coalition for Epidemic Preparedness Innovations) is in phase 1/2 [72]. COVIVAC, Newcastle Disease Virus (NDV) expressing membrane-anchored pre-fusion-stabilized trimeric SARS-CoV-2 S protein ± adjuvant CpG 1018, developed by Institute of Vaccines and Medical Biologicals, Vietnam is in phase1/2 and NDV-HXP-S; A Live Recombinant Newcastle Disease Virus-vectored COVID-19 Vaccine, developed by Sean Liu, Icahn School of Medicine at Mount Sinai is in phase 1 [7].

**Fig. 3:**
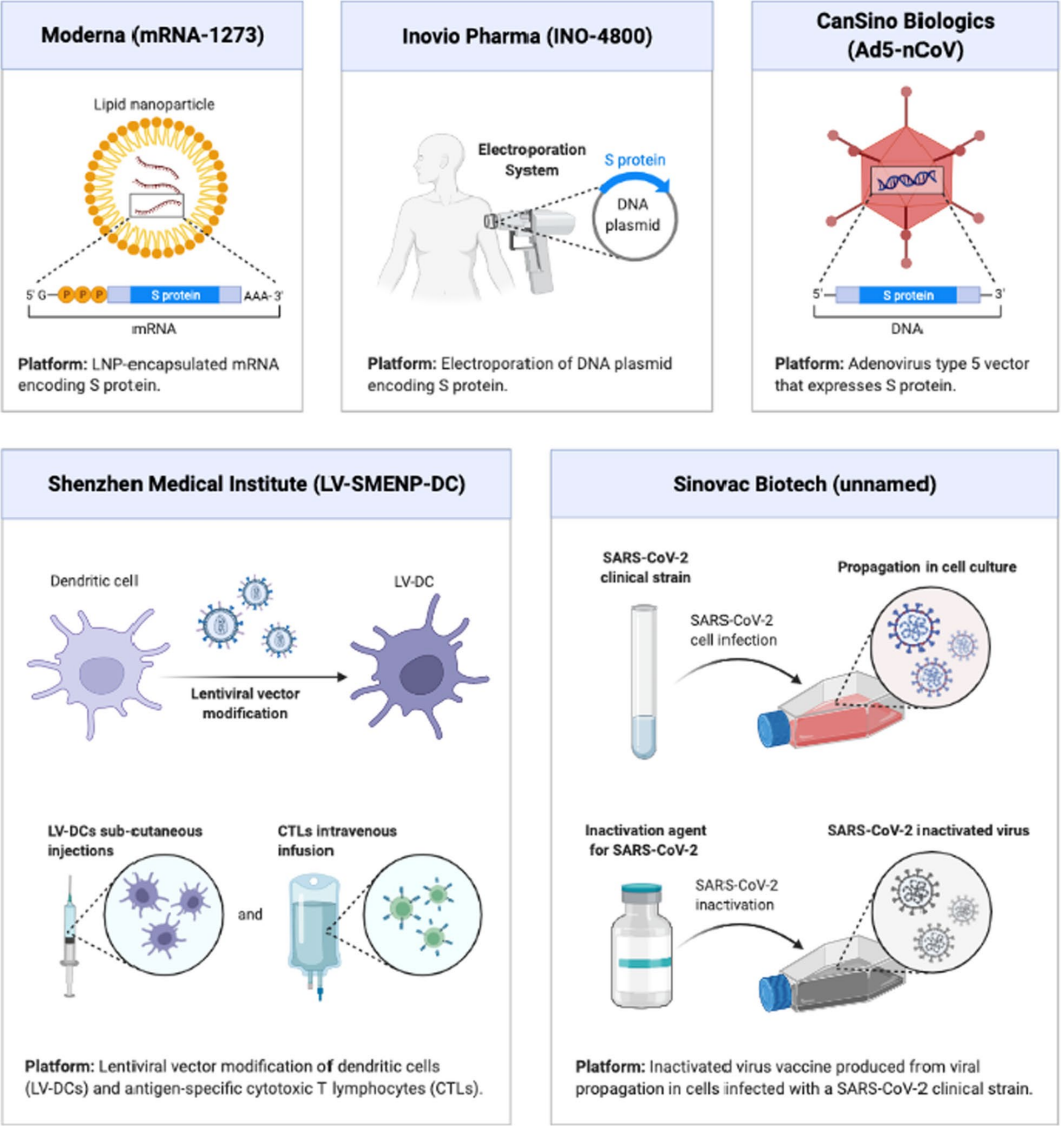
Some of the clinical phase vaccine candidates for COVID-19 (Reprinted from “Clinical Phase Vaccine Candidates for COVID-19”, by BioRender, April 2020, retrieved from https://app.biorender.com/biorender-templates/figures/5e99f5395fd61e0028682c01/t-5ea83be2d3420200adea0b6a-clinical-phase-vaccine-candidates-for-covid-19 Copyright 2022 by BioRender.)

Next-generation vaccines do not mandate the viral particle and can be formed merely based on the sequence of antigenic viral proteins. The material in the vaccine that provides data about the protein coding sequence generates its biosynthesis and, as a result, an immune response. Vaccines based on viral vectors, nucleic acids, or antigen-presenting cells are examples of next-generation vaccines [7, 73–75] (Fig. 3, Table 1).

**Table 1:** Various platforms of COVID-19 vaccines, types of vaccines, manufacturers and the phases it approached

Vaccine Platforms	Type of vaccines	Developers	Phases
Inactivated viruses	VLA2001	Valneva, National Institute for Health Research, United Kingdom	III
	TURKOVAC,	Erciyes University and the Health Institute Of Turkey (TUSEV)	III
	CovIral-Varkat	Shifa Pharmed Industrial Co	II/III
	Vero cell, BBIBP-CorV	Sinopharm	IV
	Inactivated (NDV-based) chimeric vaccine with or without the adjuvant CpG 1018	The Government Pharmaceutical Organization (GPO); PATH; Dynavax	I/II
	Inactivated COVID-19 vaccine	KM Biologics Co., Ltd	II/III
Live attenuated virus	COVI-VAC	Codagenix/Serum Institute of India	III
	MV-014-212	Meissa Vaccines, Inc	I
DNA based vaccines	COVIGEN	University of Sydney, Bionet Co., Ltd Technovalia	I
	COVID-eVax	Takis + Rottapharm Biotech	I/II
	INO4800 + electropor-ation	Inovio Pharmaceuticals + International Vaccine Institute + Advaccine	III
	AG0301-COVID19	AnGes + Takara Bio + Osaka University	II/III
	CORVax-12	OncoSec Immunotherapies; Providence Health & Services	I
	GX-19N	Genexine Consortium	II/III
	GLS-5310	Gene One Life Science, Inc	I/II
Protein subunit	VAT00008	Sanofi Pasteur + GSK	III
	Recombinant SARS-CoV-2 Fusion Protein Vaccine (V-01)	Livzon Pharmaceutical	III
	Recombinant SARS-CoV-2 vaccine (CHO Cell)	Institute of Microbiology, Chinese Academy of Sciences, AnhuiZhifeiLongcom Biopharmaceutical	III
	CIGB-66 (RBD + aluminium hydroxide)	Center for Genetic Engineering and Biotechnology (CIGB)	III
	RecombinantSars-CoV-2 Spike protein, Aluminumadjuvanted (Nanocovax)	Nanogen Pharmaceutical Biotechnology	III
	EpiVac Corona	Federal Budgetary Research Institution State Research Center of Virology and Biotechnology "Vector"	III
RNA based vaccines	mRNA-1273, mRNA-1273.351	Moderna + National Institute of Allergy and Infectious Diseases (NIAID)	IV
	BNT162b2 (3 LNP-mRNAs), also known as "Comirnaty"	Pfizer/BioNTech + Fosun Pharma	IV
	CVnCoV Vaccine	CureVac AG	III
	ARCT-021	Arcturus Therapeutics	II
	LNP-nCoVsaRNA	Imperial College London	I
	Chula Cov19 mRNA vaccine	Chulalongkorn University	I
	SARS-CoV-2 mRNA vaccine (ARCoV)	Academy of Military Science (AMS), Walvax Biotechnology and Suzhou Abogen Biosciences	III
Viral vector (non-replicating)	Recombinant novel coronavirus vaccine(Adenovirus type 5 vector)	CanSino Biological Inc./Beijing Institute of Biotechnology	IV
	Sputnik V (Gam-COVID-Vac) Adeno-based (rAd26-S + rAd5-S)	Gamaleya Research Institute; Health Ministry of the Russian Federation	III
	Ad26.COV2.S	Janssen Pharmaceutical	IV
	GRAd-COV2 (Replication defective Simian Adenovirus (GRAd encoding S)	ReiThera + Leukocare + Univercells	II/III
	VXA-CoV2-1 Ad5 adjuvanted Oral Vaccine platform	Vaxart	II
	MVA-SARS-2-S	University of Munich (Ludwig-Maximilians)	I
Viral Vectors (Replicating)	DelNS1-2019-nCoV-RBD-OPT1 (Intranasalflu- based-RBD)	University of Hong Kong, Xiamen University and Beijing Wantai Biological Pharmacy	III
	rVSV-SARS-CoV-2-S Vaccine	Israel Institute for Biological Research	II/III
Virus like particle	Corona virus- Like Particle COVID-19 (CoVLP)	Medicago Inc	III
	RBD SARS-CoV-2 HBsAg VLP vaccine	Serum Institute of India + Accelagen Pty + SpyBiotech	I/II
	VBI-2902a	VBI Vaccines Inc	I/II
	SARS-CoV-2 VLP Vaccine	The Scientific and Technological Research Council of Turkey	II
	ABNCoV2 capsid virus-like particle (cVLP) ± adjuvant MF59	Radboud University	I

The data is taken from the site of WHO vaccines in clinical development https://www.who.int/teams/blueprint/covid-19/covid-19-vaccine-tracker-and-landscape

## Conclusions

The newborn outbreak of the SARS COVID-2 has been causing a serious number of mortality and morbidity in humans. Various technology platforms are under investigation and advancement to create a competent vaccine against COVID-19. The majority of COVID-19 vaccine candidates in clinical trials are primarily targeted on the viral spike protein and its variants as the primary antigen of COVID-19 infection. Subunit vaccines, inactivated vaccines, nucleic-acid-based DNA or mRNA vaccines, and viral vectored vaccinations are the most common vaccine options based on the S-antigen. It is apparent that no single vaccine winner will be there; diverse technologies and platforms can extend divergent effectiveness and relevance in discrete epidemiological contexts. Following the recent declaration of vaccine potency against COVID-19 in clinical studies by various manufacturers for protection against disease severity, an extensive post-efficacy strategy for the next steps to ensure global inoculation is already required. There are still many scientific concerns about vaccinations that need to be solved in order to improve vaccine efficacy, such as booster dosages, vaccination regime optimization, vaccine competence, correlates of protection, the function of various available diagnostic and therapeutic countermeasures against latest variant also the increased oversight. The convenient and co-ordinated administration of these posts-efficacy burdens will lead the pandemic in a dynamic, and efficient, close. An effective vaccine is universally attributed in reducing the spread, severity and is crucial in preventing further morbidity and mortality. Although a few countries may deploy COVID-19 vaccines based purely on protection system and immunogenicity data, the purpose of vaccine development is to achieve solid evidence of vaccine efficacy to protect humans against COVID-19, enabling for the scale-up of efficient vaccine production.

## Data Availability

Not applicable.

## Abbreviations

ORFs: Open reading frames
UTRs: Unstranslated regions
PP1a: Polyprotein 1a
Nsp1-16: Non-structural proteins
sgRNAs: Subgenomic RNAs
Nsps: Non-structural proteins
LAV: Live attenuated vaccine
RBD: Receptor binding domain
MERS‐CoV: Middle East respiratory syndrome coronavirus
TMPRSS2: Transmembrane serine protease 2
rAd26: Recombinant adenovirus type 26
VLPs: Virus-like particles
GBVs: Gene- based vaccines
tPA: Plasminogen activator
ADE: Antibody-dependent enhancement
ChAd: Chimpanzee adenovirus
CMV: Cytomegalovirus
BGH: Promoter and a bovine growth hormone

## References

[1] Sultana J, Crisafulli S, Gabbay F, Lynn E, Shakir S, Trifirò G (2020). Challenges for drug repurposing in the COVID-19 pandemic era. Front Pharmacol.

[2] Chung JY, Thone MN, Kwon YJ (2021). COVID-19 vaccines: the status and perspectives in delivery points of view. Adv Drug Deliv Rev.

[3] Dos Santos WG (2020). Natural history of COVID-19 and current knowledge on treatment therapeutic options. Biomed Pharmacother.

[4] Rauch S, Jasny E, Schmidt KE, Petsch B (2018). New vaccine technologies to combat outbreak situations. Front Immunol.

[5] Volz E, Mishra S, Chand M, Barrett JC, Johnson R, Geidelberg L, Ferguson NM (2021). Assessing transmissibility of SARS-CoV-2 lineage B. 1.1. 7 in England. Nature.

[6] WHO. Classification of omicron (B.1.1.529): SARS-CoV-2 variant of concern. https://www.who.int/news/item/26-11-2021-classification-of-omicron-(b.1.1.529)-sars-cov-2-variant-of-concernStatement. 26 November 2021

[7] https://www.who.int/teams/blueprint/covid-19/covid-19-vaccine-tracker-and-landscape

[8] García-Sastre A, Mena I (2013). Novel vaccine strategies against emerging viruses. Curr Opin Virol.

[9] Wilhelm A, Widera M, Grikscheit K, Toptan T, Schenk B, Pallas C, Ciesek S (2021) Reduced neutralization of SARS-CoV-2 omicron variant by vaccine sera and monoclonal antibodies. MedRxiv

[10] Cele S, Jackson L, Khoury DS, Khan K, Moyo-Gwete T, Tegally H, Hanekom W (2021). SARS-CoV-2 Omicron has extensive but incomplete escape of Pfizer BNT162b2 elicited neutralization and requires ACE2 for infection. MedRxiv.

[11] Lurie N, Saville M, Hatchett R, Halton J (2020). Developing COVID-19 vaccines at pandemic speed. N Engl J Med.

[12] Woo PCY, Huang Y, Lau SKP, Yuen KY (2010). Coronavirus genomics and bioinformatics analysis. Viruses.

[13] Ortiz-Prado E, Simbaña-Rivera K, Gómez-Barreno L, Rubio-Neira M, Guaman LP, Kyriakidis NC, López-Cortés A (2020). Clinical, molecular, and epidemiological characterization of the SARS-CoV-2 virus and the Coronavirus Disease 2019 (COVID-19), a comprehensive literature review. Diagn Microbiol Infect Dis.

[14] Tai W, He L, Zhang X, Pu J, Voronin D, Jiang S, Jiang L (2020). Characterization of the receptor-binding domain (RBD) of 2019 novel coronavirus: implication for development of RBD protein as a viral attachment inhibitor and vaccine. Cell Mol Immunol.

[15] McBride R, van Zyl M, Fielding BC (2014). The coronavirus nucle- ocapsid is a multifunctional protein. Viruses.

[16] Yoshimoto FK (2020). The proteins of severe acute respiratory syndrome coronavirus-2 (SARS CoV-2 or n-COV19), the cause of COVID-19. Protein J.

[17] Venkatagopalan P, Daskalova SM, Lopez LA, Dolezal KA, Hogue BG (2015). Coronavirus envelope (E) protein remains at the site of assembly. Virology.

[18] Schoeman D, Fielding BC (2019). Coronavirus envelope protein: current knowledge. Virol J.

[19] Baglivo M, Baronio M, Natalini G, Beccari T, Chiurazzi P, Fulcheri E, Bertelli M (2020). Natural small molecules as inhibitors of coronavirus lipid-dependent attachment to host cells: a possible strategy for reducing SARS-COV-2 infectivity?. Acta Bio Med Atenei Parmensis.

[20] Michel CJ, Mayer C, Poch O, Thompson JD (2020). Characterization of accessory genes in coronavirus genomes. Virol J.

[21] Romano M, Ruggiero A, Squeglia F, Maga G, Berisio R (2020). A structural view of SARS-CoV-2 RNA replication machinery: RNA synthesis, proofreading and final capping. Cells.

[22] Graham RL, Sparks JS, Eckerle LD, Sims AC, Denison MR (2008). SARS coronavirusreplicase proteins in pathogenesis. Virus Res.

[23] Shang J, Ye G, Shi K, Wan Y, Luo C, Aihara H, Li F (2020). Structural basis of receptor recognition by SARS-CoV-2. Nature.

[24] Wrapp D, Wang N, Corbett KS, Goldsmith JA, Hsieh CL, Abiona O, McLellan JS (2020). Cryo-EM structure of the 2019-nCoV spike in the prefusion conformation. Science.

[25] Liu DX, Fung TS, Chong KKL, Shukla A, Hilgenfeld R (2014). Accessory proteins of SARS-CoV and other coronaviruses. Antiviral Res.

[26] Xue B, Blocquel D, Habchi J, Uversky AV, Kurgan L, Uversky VN, Longhi S (2014). Structural disorder in viral proteins. Chem Rev.

[27] Alsaadi EAJ, Jones IM (2019). Membrane binding proteins of coronaviruses. Future Virol.

[28] Tang C, Deng Z, Li X, Yang M, Tian Z, Chen Z, Chen Z (2020). Helicase of type 2 porcine reproductive and respiratory syndrome virus strain HV reveals a unique structure. Viruses.

[29] Müller C, Schulte FW, Lange-Grünweller K, Obermann W, Madhugiri R, Pleschka S, Grünweller A (2018). Broad-spectrum antiviral activity of the eIF4A inhibitor silvestrol against corona-and picornaviruses. Antiviral Res.

[30] Ehrenfeld E, Modlin J, Chumakov K (2009). Future of polio vaccines. Expert Rev Vaccines.

[31] Barrett PN, Mundt W, Kistner O, Howard MK (2009). Vero cell platform in vaccine production: moving towards cell culture-based viral vaccines. Expert Rev Vaccines.

[32] Sanders B, Koldijk M, Schuitemaker H (2015) Inactivated viral vaccines. In: Vaccine analysis: strategies, principles, and control. Springer, Berlin pp 45–80

[33] Gao Q, Bao L, Mao H, Wang L, Xu K, Yang M, Qin C (2020). Development of an inactivated vaccine candidate for SARS-CoV-2. Science.

[34] Wang H, Zhang Y, Huang B, Deng W, Quan Y, Wang W, Yang X (2020). Development of an inactivated vaccine candidate, BBIBP-CorV, with potent protection against SARS-CoV-2. Cell.

[35] Zhang J, Zeng H, Gu J, Li H, Zheng L, Zou Q (2020). Progress and prospects on vaccine development against SARS-CoV-2. Vaccines.

[36] Wang J, Peng Y, Xu H, Cui Z, Williams RO (2020). The COVID-19 vaccine race: challenges and opportunities in vaccine formulation. AAPS PharmSciTech.

[37] Hansson M, Nygren PAK, Ståhl S (2000). Design and production of recombinant subunit vaccines. Biotechnol Appl Biochem.

[38] Choi J, Kim MG, Oh YK, Kim YB (2017). Progress of Middle East respiratory syndrome coronavirus vaccines: a patent review. Expert Opin Ther Pat.

[39] Salvatori G, Luberto L, Maffei M, Aurisicchio L, Roscilli G, Palombo F, Marra E (2020). SARS-CoV-2 SPIKE PROTEIN: an optimal immunological target for vaccines. J Transl Med.

[40] Hoffmann M, Kleine-Weber H, Schroeder S, Krüger N, Herrler T, Erichsen S, Pöhlmann S (2020). SARS-CoV-2 cell entry depends on ACE2 and TMPRSS2 and is blocked by a clinically proven protease inhibitor. Cell.

[41] Chauhan G, Madou MJ, Kalra S, Chopra V, Ghosh D, Martinez-Chapa SO (2020). Nanotechnology for COVID-19: therapeutics and vaccine research. ACS Nano.

[42] Huang C, Wang Y, Li X, Ren L, Zhao J, Hu Y, Cao B (2020). Clinical features of patients infected with 2019 novel coronavirus in Wuhan, China. Lancet.

[43] Lin LY, Tran TH (2020). Coronaviruses pandemics: can neutralizing antibodies help?. Life Sci.

[44] He Y, Zhou Y, Liu S, Kou Z, Li W, Farzan M, Jiang S (2004). Receptor-binding domain of SARS-CoV spike protein induces highly potent neutralizing antibodies: implication for developing subunit vaccine. Biochem Biophys Res Commun.

[45] Tai W, Zhao G, Sun S, Guo Y, Wang Y, Tao X, Zhou Y (2016). A recombinant receptor-binding domain of MERS-CoV in trimeric form protects human dipeptidyl peptidase 4 (hDPP4) transgenic mice from MERS-CoVinfection. Virology.

[46] Liu J, Sun Y, Qi J, Chu F, Wu H, Gao F, Gao GF (2010). The membrane protein of severe acute respiratory syndrome coronavirus acts as a dominant immunogen revealed by a clustering region of novel functionally and structurally defined cytotoxic Tlymphocyteepitopes. J Infect Dis.

[47] Keech C, Albert G, Cho I, Robertson A, Reed P, Neal S, Glenn GM (2020). Phase 1–2 trial of a SARS-CoV-2 recombinant spike protein nanoparticle vaccine. N Engl J Med.

[48] Liang JG, Su D, Song TZ, Zeng Y, Huang W, Wu J, Liang P (2021). STrimer, a COVID-19 subunit vaccine candidate, induces protective immunity in nonhuman primates. Nat Commun.

[49] Pandey SC, Pande V, Sati D, Upreti S, Samant M (2020). Vaccination strategies to combat novel corona virus SARS-CoV-2. Life Sci.

[50] Logunov DY, Dolzhikova IV, Zubkova OV, Tukhvatullin AI, Shcheblyakov DV, Dzharullaeva AS, Gintsburg AL (2020). Safety and immunogenicity of an rAd26 and rAd5 vector-based heterologous prime-boost COVID-19 vaccine in two formulations: two open, non-randomised phase 1/2 studies from Russia. Lancet.

[51] Zeltins A (2013). Construction and characterization of virus-like particles: a review. Mol Biotechnol.

[52] Dai S, Wang H, Deng F (2018). Advances and challenges in enveloped virus-like particle (VLP)-based vaccines. J Immunol Sci.

[53] Prasad A, Muthamilarasan M, Prasad M (2020). Synergistic antiviral effects against SARS-CoV-2 by plant-based molecules. Plant Cell Rep.

[54] Gurunathan S, Wu CY, Freidag BL, Seder RA (2000). DNA vaccines: a key for inducing long-term cellular immunity. Curr Opin Immunol.

[55] Kutzler MA, Weiner DB (2008). DNA vaccines: ready for prime time?. Nat Rev Genet.

[56] Flingai S, Czerwonko M, Goodman J, Kudchodkar SB, Muthumani K, Weiner DB (2013). Synthetic DNA vaccines: improved vaccine potency by electroporation and codeliveredgenetic adjuvants. Front Immunol.

[57] Vogel AB, Lambert L, Kinnear E, Busse D, Erbar S, Reuter KC, Tregoning JS (2018). Self-amplifying RNA vaccines give equivalent protection against influenza to mRNA vaccines but at much lower doses. Mol Ther.

[58] Mahase E (2020). Covid-19: UK approves Pfizer and BioNTech vaccine with rollout due to start next week. BMJ.

[59] Pardi N, Hogan MJ, Porter FW, Weissman D (2018). mRNA vaccines—a new era invaccinology. Nat Rev Drug Discov.

[60] Mulligan MJ, Lyke KE, Kitchin N, Absalon J, Gurtman A, Lockhart S, Jansen KU (2020). Phase I/II study of COVID-19 RNA vaccine BNT162b1 in adults. Nature.

[61] Ura T, Okuda K, Shimada M (2014). Developments in viral vector-based vaccines. Vaccines.

[62] Sebastian S, Lambe T (2018). Clinical advances in viral-vectored influenza vaccines. Vaccines.

[63] Waehler R, Russell SJ, Curiel DT (2007). Engineering targeted viral vectors for gene therapy. Nat Rev Genet.

[64] Zhu FC, Li YH, Guan XH, Hou LH, Wang WJ, Li JX, Chen W (2020). Safety, tolerability, and immunogenicity of a recombinant adenovirus type-5 vectored COVID-19 vaccine: a dose-escalation, open-label, non-randomised, first-in-human trial. Lancet.

[65] van Doremalen N, Lambe T, Spencer A, Belij-Rammerstorfer S, Purushotham JN, Port JR, Munster VJ (2020). ChAdOx1 nCoV-19 vaccine prevents SARS-CoV-2 pneumonia in rhesus macaques. Nature.

[66] Zhang H, Penninger JM, Li Y, Zhong N, Slutsky AS (2020). Angiotensin-converting enzyme 2 (ACE2) as a SARS-CoV-2 receptor: Molecular mechanisms and potential therapeutic target. Intensive Care Med.

[67] Silva-Cayetano, A., Foster, W. S., Innocentin, S., Belij-Rammerstorfer, S., Spencer, A. J., Burton, O. T., Linterman, M. A. (2021). A booster dose enhances immunogenicity of theCOVID-19 vaccine candidate ChAdOx1 nCoV-19 in aged mice.*Med*, *2*(3), 243–262. 10.1016/j.medj.2020.12.006 PMC7833318 33521747

[68] Callaway E (2020). Russia's fast-track coronavirus vaccine draws outrage over safety. Nature.

[69] https://sputnikvaccine.com/newsroom/pressreleases/second-interim-analysis-of-clinical-trialdata-showed-a-91-4-efficacy-for-the-sputnik-v-vaccine-on-d/

[70] Du L, He Y, Zhou Y, Liu S, Zheng BJ, Jiang S (2009). The spike protein of SARS-CoV—a target for vaccine and therapeutic development. Nat Rev Microbiol.

[71] Zhu FC, Guan XH, Li YH, Huang JY, Jiang T, Hou LH, Chen W (2020). Immunogenicity and safety of a recombinant adenovirus type-5-vectored COVID-19 vaccine in healthy adults aged 18 years or older: a randomised, double-blind, placebo-controlled, phase 2 trial. Lancet.

[72] Karpiński TM, Ożarowski M, Seremak-Mrozikiewicz A, Wolski H, Wlodkowic D (2021). The 2020 race towards SARS-CoV-2 specific vaccines. Theranostics.

[73] Soema PC, Kompier R, Amorij JP, Kersten GF (2015). Current and next generation influenza vaccines: formulation and production strategies. Eur J Pharm Biopharm.

[74] van Riel D, de Wit E (2020). Next-generation vaccine platforms for COVID-19. Nat Mater.

[75] Wallis J, Shenton DP, Carlisle RC (2019). Novel approaches for the design, delivery and administration of vaccine technologies. Clin Exp Immunol.

